# Strontium-Doped Calcium Phosphate and Hydroxyapatite Granules Promote Different Inflammatory and Bone Remodelling Responses in Normal and Ovariectomised Rats

**DOI:** 10.1371/journal.pone.0084932

**Published:** 2013-12-23

**Authors:** Carina Cardemil, Ibrahim Elgali, Wei Xia, Lena Emanuelsson, Birgitta Norlindh, Omar Omar, Peter Thomsen

**Affiliations:** 1 Department of Biomaterials, Institute of Clinical Sciences, Sahlgrenska Academy at University of Gothenburg, Gothenburg, Sweden; 2 Department of Oral and Maxillofacial Surgery, Örebro University Hospital, Örebro, Sweden; 3 Applied Materials Science, Department of Engineering Sciences, Uppsala University, Uppsala, Sweden; 4 BIOMATCELL VINN Excellence Center of Biomaterials and Cell Therapy, Gothenburg, Sweden; Institute for Bioengineering of Catalonia, Spain

## Abstract

The healing of bone defects may be hindered by systemic conditions such as osteoporosis. Calcium phosphates, with or without ion substitutions, may provide advantages for bone augmentation. However, the mechanism of bone formation with these materials is unclear. The aim of this study was to evaluate the healing process in bone defects implanted with hydroxyapatite (HA) or strontium-doped calcium phosphate (SCP) granules, in non-ovariectomised (non-OVX) and ovariectomised (OVX) rats. After 0 (baseline), six and 28d, bone samples were harvested for gene expression analysis, histology and histomorphometry. Tumour necrosis factor-α (TNF-α), at six days, was higher in the HA, in non-OVX and OVX, whereas interleukin-6 (IL-6), at six and 28d, was higher in SCP, but only in non-OVX. Both materials produced a similar expression of the receptor activator of nuclear factor kappa-B ligand (RANKL). Higher expression of osteoclastic markers, calcitonin receptor (CR) and cathepsin K (CatK), were detected in the HA group, irrespective of non-OVX or OVX. The overall bone formation was comparable between HA and SCP, but with topological differences. The bone area was higher in the defect centre of the HA group, mainly in the OVX, and in the defect periphery of the SCP group, in both non-OVX and OVX. It is concluded that HA and SCP granules result in comparable bone formation in trabecular bone defects. As judged by gene expression and histological analyses, the two materials induced different inflammatory and bone remodelling responses. The modulatory effects are associated with differences in the spatial distribution of the newly formed bone.

## Introduction

Bone tissue has an inheritable self-healing capacity. However, this may fail in some situations where the deficiency is too large to be regenerated spontaneously. The process may be further hindered when the bone microstructure is compromised due to systemic diseases, e.g. osteoporosis. These situations traditionally require augmentation procedures where auto- or allografts represent the gold standard. However, the limited material supply, donor-site morbidity, transmission of infectious diseases and the risk of immunological rejection are still drawbacks [1]. It is therefore a need to develop new or to modify existing materials, which further augment and/or accelerate bone healing and regeneration. 

Synthetic hydroxyapatite and beta tricalcium phosphate resemble the mineral phase of bone to varying extents. Chemical composition, microstructure and crystallinity could be contributory factors determining the biological responses to these materials. In addition, it has been suggested that the incorporation of bioactive ions, e.g. sodium, carbonate, magnesium and strontium, can improve the biological performance of calcium phosphates [2-4]. In addition to their biological roles in bone metabolism, these ions may provide physicochemical modifications in the produced material, which can have a favourable effect on the bone response [5]. Strontium has been indicated to improve bone strength and provide beneficial effects in patients with osteoporosis [6]. Although strontium-incorporated apatite may provide a promising bone substitute, *in vivo* studies are needed to evaluate the bone response to such materials, preferentially under compromised conditions.

The ovariectomised (OVX) rat model is widely used to simulate compromised bone conditions in osteoporosis resulting in reduced bone mineral density and deranged bone microarchitecture [7,8]. Experimental studies have shown that estrogen deficiency due to OVX negatively affects fracture healing and the osseointegration of titanium implants [9,10]. It has also been shown that factors governing the different stages of bone healing are affected, locally or systemically, in osteoporotic conditions [11-13]. The systemic administration of strontium has been reported to reduce bone resorption, maintain high bone formation and promote fracture healing in OVX rats [14-16]. Although the exact mechanism of action by strontium on bone events has not yet been established, many hypotheses have been presented. For instance, *in vitro* studies revealed that osteoblasts from OVX rats release lower levels of IL-6 when cultured on strontium-substituted HA compared with those cultured on HA [17]. With respect to bone formation and remodelling, strontium has been suggested to have a dual effect, i.e. pro-osteogenic and anti-osteoclastic [6,16,18-20]. *In vivo*, the systemic administration of strontium in OVX rats resulted in increased bone formation and less osteoclast surface in the proximal tibia [18].

These previous data corroborate the complexity of the mechanisms in bone-related cellular activities when exposed to strontium. The way these mechanisms influence the healing of bone defects filled with calcium phosphate biomaterials, with or without strontium substitution, needs to be evaluated. The aim of this study was to evaluate the differences in biological response to hydroxyapatite (HA) and strontium-doped calcium phosphate (SCP) granules in both normal and compromised bone conditions at two different time points. We hypothesised that factors involved in bone healing are regulated differently in response to HA and SCP granules, thereby affecting the bone regeneration pattern and rate in trabecular bone defects.

## Materials and Methods

### 1 Granule preparation and characterisation

Analytical grade calcium chloride, strontium nitrate, sodium chloride, magnesium chloride, potassium chloride, sodium phosphate dibasic and potassium phosphate monobasic were all obtained from Sigma-Aldrich (New York, USA). From these, SCP and HA particles were prepared which were subsequently molded as SCP and HA granules. 

#### 1.1 Particle preparation

Strontium-doped calcium phosphate particles were synthesized using a surfactant-free mineralisation method [21]. Strontium nitrate was dissolved in PBS with concentrations of Ca (0.9 mM) and phosphates (10 mM). The initial pH was 7.4. The obtained solution was put into a tightly covered glass bottle at 100°C for 24h and then centrifuged. The precipitate separated from the solution was washed with ethanol and dried at 60°C for one day. HA particles were prepared by a precipitation method. Calcium nitrate solution (1.1 mM) and ammonium phosphate dibasic (0.66 mM) solution were mixed and the Ca/P ratio was fixed at 1.67. The pH of the solution was adjusted to 11 using ammonia solution. The aqueous mixture was stored in a Teflon container at 150°C for 24h. The precipitate separated from the solution was washed with ethanol several times and dried at 60°C for one day. 

#### 1.2 Granule preparation

A granule model, a Teflon plate with holes in diameter of 1.5 mm and height of 1.5 mm, was used to produce SCP and HA granules. First of all, SCP and HA particles were mixed with water in a particle/water weight ratio of 0.45 - 0.5. In order to adjust the viscosity of the obtained SCP and HA pastes, 10 wt% of calcium phosphate cement, containing 55 wt% of beta-tricalcium phosphate and 45 wt% of monocalcium phosphate, was added to the SCP and HA pastes. The obtained pastes were poured into the models and set at 37°C at 100% humidity for two days. Then the column-shaped granules were removed from the model. The size of the obtained granules was the same as the size of holes in Teflon model. The as-prepared granules were calcined at 600°C for one hour to improve their mechanical strength. The prepared granules were autoclaved at 125°C and delivered sterile in glass vials.

#### 1.3 Characterization

X-ray diffraction (XRD) analysis of particles and granules was conducted on a Siemens Diffractometer 5000 with Cu (Kα) radiation at an operating condition of 40 kV and 40 mA. Crystal phase identification was determined by the database from ICDD (International Centre for Diffraction Data). 

The amount of different ions in each granule type as well as the level of strontium substitution was determined by inductively coupled plasma atomic emission spectroscopy (ICP-AES). In brief, the granules were dissolved in 1 M HCl solution and analysed by ICP-AES. The level of strontium substitution was determined by calculating the Sr/Ca ratio. The data describing the amount of elements in each granule type (HA and SCP) as well as the Sr/Ca ratio in SCP granules is provided as Table S1. 

The porosity of each granule type (HA and SCP; n = 5) was determined using micro computed tomography. The analysis showed that the porosity was 0.41 ± 0.19 and 0.46 ± 0.18, respectively.

The morphology was analyzed in a field-emission scanning electron microscope (FESEM, LEO 1550) working at 5 kV.

### 2 In vitro degradation of strontium calcium phosphate (SCP) and hydroxyapatite (HA) granules

The *in vitro* degradation of SCP and HA granules was performed in Dulbecco’s phosphate buffered saline containing calcium and magnesium (DPBS), with an inorganic composition close to that of simulated body fluid. Thirty granules were soaked in 10 ml of DPBS and put on a horizontal shaker up to 28d. The weight loss of granules was evaluated by collecting the rest of the granules in the containers. In addition, a release analysis was performed in order to evaluate the release of strontium, calcium, phosphate and magnesium over time. In brief, thirty granules were soaked in 10 ml of DPBS and put on a horizontal shaker for different time points (n = 3 for each granule type and time point). The surrounding medium was collected, after 3, 7, 14 and 28d, and analysed using ICP-AES. 

### 3 Experimental design and animal model

#### 3.1 Ethics statement

The animal experiments were approved by the University of Gothenburg Local Ethics Committee for Laboratory Animals (dnr 279/2011). Implantation procedure was performed under inhalation general anaesthesia and local injection of xylocaine. Furthermore, all animals received subcutaneous injection of analgesic in order to minimize the post-operative pain.

#### 3.2 Pre-test in polyurethane foam

The amount of the two materials needed to fill a comparable defect size in the animal model was estimated by filling holes created in solid polyurethane foams (Sawbones^®^, Pacific Research Laboratories, Vashon, United States). The HA and SCP granules were inserted in five holes for each material, created in the polyurethane foam, using a trephine (2.3 mm diameter; 3 mm depth). The granules were either inserted in dry holes or in holes which had received a single drop of water. After two hours the materials were collected. The granules in the dry holes amounted to a total of six granules per defect. In the holes where a drop of water had been inserted prior to the granules, a mean of 11 granules were inserted. There was no difference between the two materials in the mean number of granules inserted in the holes. The mean weight of one HA and SCP granule was 3.6 and 2.4 mg, respectively.

#### 3.3 Animal surgery

Sixty-four, four-month-old female Sprague-Dawley rats (Charles River, Sulzfeld, Germany) were used. Half (32 rats) were ovariectomised (OVX) at 12 weeks of age, keeping 32 rats non-ovariectomised (non-OVX). Rats were allowed a three-week acclimatization and free movement with food and water *ad libitum*. At surgery, the mean weights were 322 and 317 g in the non-OVX and OVX groups, respectively. The animals were anaesthetised using isofluorane (Isoba Vet, Schering-Plough, Uxbridge, UK) gas inhalation using an anaesthesia unit (Univentor 400, Zejtun, Malta). Anaesthesia was maintained by continuous administration of isofluorane via a mask. The distal aspect of the femur was cleaned with 5 mg/mL of chlorhexidine in 70% ethanol. The leg was shaved and a longitudinal incision was made, followed by skin and periosteal reflection. A defect was made in each femur using a 2.3 mm-diameter trephine under profuse irrigation with NaCl 0.9%. The bone harvested from the defect site was collected from the trephine and preserved for the determination of steady-state gene expression (baseline; n = 8). The HA granules were then placed in one defect and SCP granules were placed in the contralateral one. Every defect received 7-11 granules. The wound was closed with s.c. resorbable polyglactin (4-0, Vicryl) and i.c. resorbable (4-0, Monocryl) sutures. Each rat received an analgesic (Temgesic 0.03 mg/kg, Reckitt & Coleman, Hull, UK) s.c. post-operatively. The retrieval procedure was performed at six and 28d (32 rats at each time point) when the animals were sacrificed using an overdose of barbiturate (Mebumal, ACO Läkemedel AB, Solna, Sweden). Before sample retrieval, blood was withdrawn from the jugular vein, under isoflurane anaesthesia, for an enzyme-linked immunosorbent assay (ELISA). The skin was then re-opened and the bone defects filled with granules were identified. On the side intended for gene expression analysis, the defect was retrieved using a 2.3 mm trephine. The samples were preserved in tubes containing RNAlater (n = 8). On the contralateral side (for histology and histomorphometry), a dental disc was used to harvest the distal end of the femur containing the filled bone defect *en bloc*. The harvested samples were then immersed in formalin (n = 8).

### 4 Enzyme-linked immunosorbent assay (ELISA)

Detailed procedures and results are provided as Supporting information (Appendix S1 and Figure S1, respectively).

### 5 Quantitative polymerase chain reaction (qPCR)

Gene expression analysis was performed at TATAA Biocenter AB, Gothenburg, Sweden. The trephine-retrieved samples were homogenised in a TissueLyser^®^ instrument using a 5 mm stainless steel bead (Qiagen GmbH, Hilden, Germany), followed by phase separation with TriZol^®^ solution (Qiagen GmbH, Hilden, Germany) during centrifugation at 12,000 g for 15 min. To extract RNA from the separated aqueous phase, an RNA Tissue Kit SII on the QuickGene extraction robot (Fujifilm Life-Science, Tokyo, Japan) was used. To reduce genomic DNA contamination, all samples were DNase treated with an RNase-free DNase Set (Qiagen GmbH, Hilden, Germany) during extraction. All RNA samples were analysed for RNA quantity using the NanoDrop ND-1000 spectrophotometer (NanoDrop Technologies, Inc., Wilmington, USA). RNA quality was measured with the Experion™ RNA StdSens Analysis Kit (Bio-Rad Laboratories, Inc., Hercules, USA) on a set of representative samples from each group. Primer design was performed with Primer3-based software [22] and qPCR assays were tested on a purified PCR product generated from cDNA of rat tissue. The following predesigned assays were purchased from TATAA Biocenter AB: tumour necrosis factor-α (TNF-α), interleukin-6 (IL-6), caspase 3, collagen type I alpha 1 (Col1a1), alkaline phosphatase (ALP), osteocalcin (OC), osteoprotegerin (OPG), receptor activator of NF-kappaB ligand (RANKL), calcitonin receptor (CR), cathepsin K (CatK) and vascular endothelial growth factor A (VEGFA). Prior to sharp analysis, a panel of reference genes was screened by analysing 20% of the samples. The expression profiles of the reference genes were evaluated by geNorm [23] and Normfinder [24] software, in order to determine the best reference genes for normalisation. The most stable expression was achieved by YHWAZ and HPRT1 and they were chosen as the reference genes. Furthermore, and before the reverse transcription, the samples were normalised to 25 ng/μl. All reverse transcriptions were performed using an iScript cDNA Synthesis Kit (Bio-Rad Laboratories, Inc., Hercules, USA). The analysis was performed on all samples with the assays targeting the 11 different mRNA transcripts and the best two selected reference genes, using a 10 μl reaction volume in duplicates on the LightCycler480 Instrument (Roche Diagnostics Corporation, Indianapolis, USA) with iQTM SYBR Green Supermix (Bio-Rad Laboratories Inc., Hercules, USA). Quantities of the target genes were normalised using the mean of Cq values of the selected reference genes. The normalised relative quantities were calculated using the delta Cq method and 90% PCR efficiency (k*1.9^∆ct^) [25].

### 6 Histology and histomorphometry

The formalin-fixed samples were dehydrated by a graded series of ethanol and embedded in acrylic resin (LR White) (London Resin Company Ltd, Berkshire, UK). The long axis of the defect was cut using a diamond saw. Ground sections were prepared using sawing and grinding (Exakt Apparatebau GmbH & Co, Norderstedt, Germany). Sections with a final thickness of 10-20 μm were stained with 1% toluidine blue. All sections were coded and evaluated blind for histology and histomorphometry using a light microscope (Nikon Eclipse E600). 

**Figure 1:**
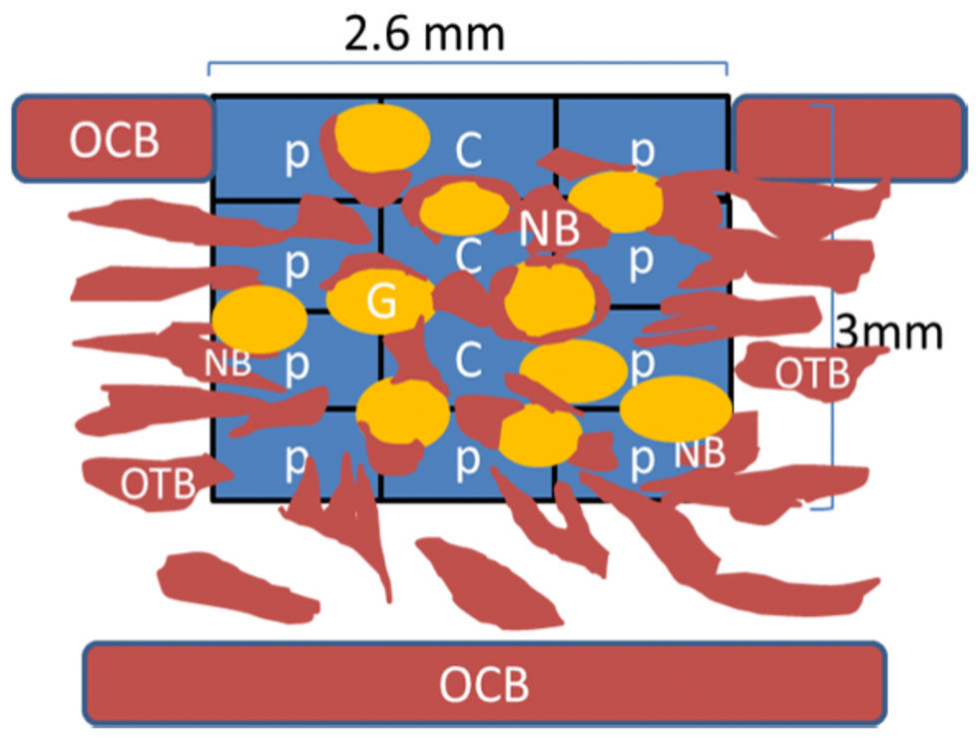
Schematic diagram showing the area of measurements (histomorphometry). A software rectangular grid consisting of twelve zones covers and determines the entire area of the treated defect. C = Central region; P = Peripheral region; OCB = Original cortical bone; OTB = Original trabecular bone; NB = new bone; G = Granule.

Histomorphometry was carried out on each section using a 10x objective. In each defect, the area percentages of granules, as well as the newly formed bone, were calculated. The measurements were performed either on the total defect area level or by dividing the total defect area into central and peripheral areas. This was achieved by performing the measurements using a software grid consisting of twelve zones, which covered the total area of defect (Figure 1). The central region consisted of the sum of the three middle zones, whereas the peripheral region was the sum of the remaining zones peripheral to the central ones. The area of the newly formed bone and the area of granules were determined separately in every zone and the area percentage of each (bone or granules) was then calculated with respect to the total defect area or to the respective region (central or peripheral).

### 7 Statistical analysis

For the ELISA results, a statistical comparison was made between the two animal types (i.e. non-OVX vs. OVX). Statistical comparisons of the histomorphometry and gene expression results were made between the two different materials (HA vs. SCP) for each animal type and between the two animal types for each material type. Furthermore, the gene expression in the defects of all the groups was compared with steady-state (baseline; BL) gene expression. Non-parametric tests were used to identify statistical differences between the groups. Wilcoxon’s signed ranks (HA vs. SCP) or Mann-Whitney (non-OVX vs. OVX) tests were used to determine the statistical difference between each two groups, respectively. Analyses were carried out using SPSS Version 10 software (SPSS, Inc., Chicago, USA) and the significance was set at *p < 0.05*. The data are presented as the mean ± standard error of the mean. 

## Results

### 1 Material characterisation

**Figure 2:**
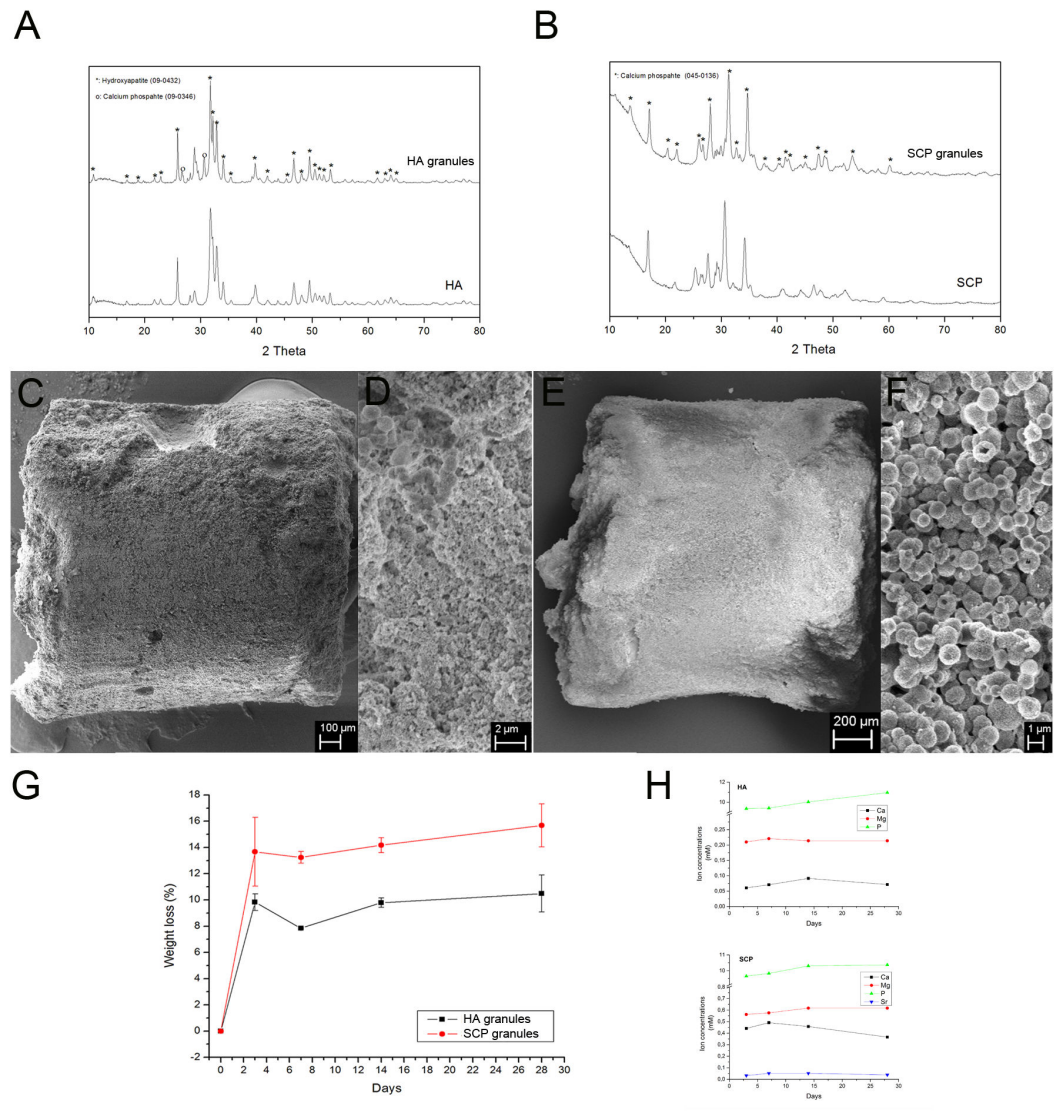
Material characterisation, *in* *vitro* degradation and ion release. (A and B) X-ray diffraction analysis (XRD) of the hydroxyapatite (HA) and the strontium substituted calcium phosphate (SCP) granules. (C - F) Scanning electron microscopy (SEM) images of the HA (C, D) and SCP (E, F) granules. (G) *In* *vitro* degradation profile of HA and SCP granules. (H) Release of Ca, P, Mg and Sr ions from HA (top) and SCP (bottom) granules during 3, 7, 14 and 28d.

XRD patterns of the HA and SCP particles and granules showed that both materials are basically composed of calcium and phosphates (Figure 2 A, B). For HA (Figure 2A), all peaks of the XRD pattern of the HA particles indicated that the synthesized particles were hydroxyapatite (09-0432). After calcination, the crystallinity of the obtained HA granules increased and a few peaks for tricalcium phosphate (09-0346) appeared (Figure 2A). For SCP (Figure 2B), the peaks of the XRD pattern of SCP particles were beta-tricalcium phosphate (04-008-8714). After calcination, the obtained SCP granules showed the same crystallinity without any new phases detected.

The SEM images show that the obtained HA and SCP granules had the same shape and granule size (Figure 2C and E, respectively). In the cross-section of an individual HA granule, the HA particles appeared random in shape with sub-micrometre size (Figure 2D). On the other hand, SCP particles appeared spherical and hollow, with a size of around 1 μm (Figure 2F). Furthermore, the cross-sectional images showed that HA particles are more densely packed than SCP particles. The SCP particles were shown to maintain the spherical shape after the calcination (Figure 2F).

### 2 In vitro degradation of HA and SCP granules

After 28d, half the SCP granules were unable to maintain the columnar shape, while 90% of the HA granules maintained their original shape. The degradation rates of the two granules gradually increased (Figure 2G). The SCP and HA granules had 15 wt% and 11 wt% loss, respectively, after 28d. The rapid weight loss after three days was probably due to the loss of loose powder on the granule surfaces. As both SCP and HA are bioactive materials, new apatite formed during soaking in a simulated body fluid, which may reduce the degradation rate. The release analysis (Figure 2H) showed the main following findings: (1) the concentration of Ca ions had a slight, but steady increase over time for both HA and SCP. (2) The concentration of P ions revealed a slight increase until 14d, for HA, and 7d, for SCP and thereafter slightly decreased for both granule types. (3) The release of SrSr ion, in the SCP group, had a slight increase from 0.03 ± 0.002 (at 3d) to 0.05 ± 0.009 (at 7d), reached a plateau of 0.05 ± 0.01 (at 14d) and slightly decreased to 0.04 ± 0.004 (at 28d). No SrSr release was detected in the HA group.

### 3 Animal model (general findings)

The trephine-created defects were filled with the HA and SCP granules. The internal diameter of the trephine was 2.3 mm. Since the metal thickness of the trephine is 0.3 mm, the defect size was 2.6 mm in diameter. The average numbers of granules inserted in the femur defects were 7.42 ± 1.29 granules (net weight 24.18 ± 5.68 mg) for HA and 9.98 ± 1.84 granules (net weight 20.92 ± 6.26 mg) for SCP. This was less than the estimated number in the pre-test in polyurethane foam. This could possibly be explained by the situation *in vivo*, where blood from the trabecular bone, together with debris that follows when the trephine cuts through the bone, is mixed with the granules in the defect. The SCP granules condensed more easily when inserted in the defect, while the HA granules appeared to keep their form, which could explain the larger number of granules needed in the SCP group. Postoperatively, macroscopic observations indicated that the sites healed uneventfully with no clinical evidence of surgical complications or infection during the experimental periods.

### 4 Gene expression analysis

Comparisons were made of gene expression in bone samples retrieved during the defect preparation (baseline; BL) and the tissue harvested from the granule-filled defects after six and 28d. In this way, factors involved in inflammation, bone formation, bone resorption, angiogenesis and apoptosis were analysed at BL and six and 28d after the insertion of HA or SCP granules.

#### 4.1 Gene expression of proinflammatory and apoptosis markers

**Figure 3:**
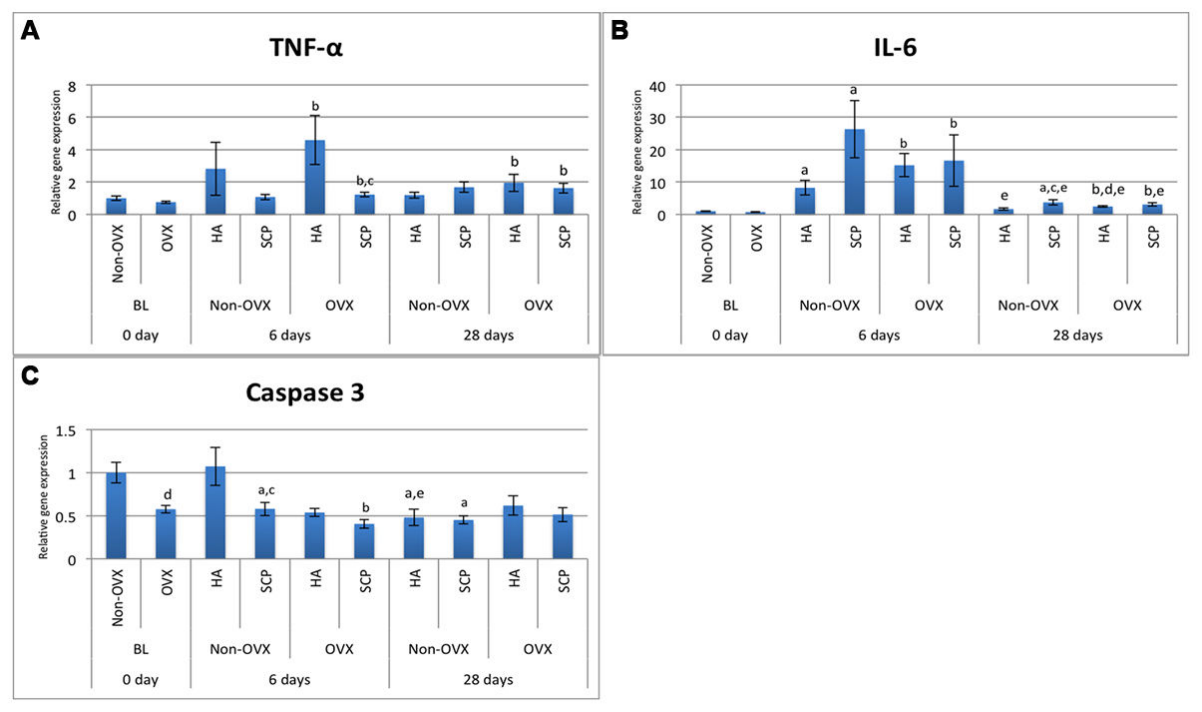
Gene expression analysis of inflammatory and apoptosis markers. The analysis was performed on the tissue harvested from defects filled with hydroxyapatite (HA) and strontium-doped calcium phosphate (SCP) in non-OVX and OVX rats after six and 28d of implantation. Expression levels of inflammatory markers TNF-α (A) and IL-6 (B) and apoptosis marker Caspase 3 (C). Statistically significant differences (p < 0.05) are indicated by the small letters: a = significant difference between baseline non-OVX and non-OVX (HA or SCP; 6 or 28d); b = significant difference between baseline OVX and OVX (HA or SCP; 6 or 28d); c = significant difference between HA and SCP (non-OVX or OVX; 6 or 28d); d = significant difference between non-OVX and OVX (HA or SCP; 6 or 28d); e = significant difference between six days and 28d (HA or SCP; non-OVX or OVX). The results are presented as the mean ± SEM.

The baseline gene expression and the temporal changes in gene expression were evaluated. In the non-OVX rats, the TNF-α expression in the HA- and SCP-filled defects did not differ significantly from BL, at either six or 28d (Figure 3A, Table 1). The IL-6 expression revealed an eight- and 26-fold higher expression in HA and SCP, respectively, after six days of healing compared with BL (Figure 3B, Table 1). After 28d, the expression of IL-6 decreased by factors of 5, in HA, and 7, in SCP, compared with the expression levels at six days (Figure 3B, Table 1). However, a 3.7-fold higher expression of IL-6 was still observed in the SCP defects at 28d compared with the BL expression (Figure 3B, Table 1). With respect to caspase 3 expression, both materials (HA and SCP) revealed a two-fold significantly lower expression at 28d compared with BL, whereas only the SCP revealed a 1.7-fold significantly lower expression when comparing six days with BL (Figure 3C, Table 1). 

**Table 1:** Fold differences in gene expression.

	**Non-OVX**						**OVX**					
	**HA**			**SCP**			**HA**			**SCP**		
	**6d^*a*^/ BL^*b*^**	**28d^*a*^/ BL^*b*^**	**28d^*a*^/ 6d^*b*^**	**6d^*a*^/ BL^*b*^**	**28d^*a*^/ BL^*b*^**	**28d^*a*^/ 6d^*b*^**	**6d^*a*^/ BL^*b*^**	**28d^*a*^/ BL^*b*^**	**28d^*a*^/ 6d^*b*^**	**6d^*a*^/ BL^*b*^**	**28d^*a*^/ BL^*b*^**	**28d^*a*^/ 6d^*b*^**
TNF-α	2.8	1.2	-2.4	1.1	1.6	1.6	6*	2.6*	-2.3	1.6*	2.1*	1.3
IL-6	8.2*	1.7*	-5*	26*	3.7*	-7.1*	20.7*	3.3*	-6.2*	22.7*	4.2*	-5.4*
Caspase3	1.1	-2.1	-2.2*	-1.7*	-2.2*	-1.3	-1.1	1.1	1.1	-1.4*	-1.1	1.3
ALP	2.5*	-3.2*	-8.2*	1.2	-3.5*	-4.4*	2.3*	1.3	-1.8	1.4	-1.1	-1.5
Col1a1	3.1*	-1.2	-3.6*	1.7	-1.2	-2.1	-1.1	1	1.1	-1.6	-1.2	1.3
OC	-3.7*	-2.8*	1.3	-5.6*	-3.1*	1.8	-5.3*	-1.4	3.9	-14 *	-2*	7.4*
OPG	3.1*	-2.2	-6.8*	1.8*	-1.7	-3*	2.3*	1.1	-2.1*	2.5*	-1.3	-3.3*
RANKL	5.5*	1.2	-4.8*	4.5*	1.1	-4.1*	6.7*	2.1*	-3.2*	7.6*	1.5	-5*
CR	3.3*	1.6	-2*	1.2	-1.7	-2*	3.8*	3.8*	1	2.3*	2	-1.2
CatK	2.5*	2*	-1.2	1.5	1.7	1.1	5.8*	9.1*	1.6*	4.4*	3.6*	-1.2
VEGFA	1.5	-2.4*	-3.5*	2.3*	-1.7	-3.9*	7.7*	1.4	-5.7*	4.7*	1.3	-3.6*

The Table shows the temporal changes between baseline (BL) and each evaluation period and between 6d and 28d, for each material and animal group.

Positive values indicate higher expression in (**^*a*^**) than in (**^*b*^**) whereas negative values indicate higher expression in (**^*b*^**) than in (**^*a*^**). Statistically significant differences are indicated by asterisks (*p < 0.05).

In the OVX rats, the TNF-α expression at six days was significantly higher, six times, in HA, and 1.6 times in SCP, compared with the BL level (Figure 3A, Table 1). The expression of IL-6 revealed a similar pattern, but with a higher fold-difference (> 20-fold) in both material groups, at six days compared with BL. From six to 28d, while a five- to six-fold reduction in the expression of IL-6 was recorded for both materials, no significant change in TNF-α expression was detected. In spite of this, at 28d, both HA and SCP groups retained a higher expression of both cytokines, ranging from two- to four-fold, compared with BL (Figure 3A, B, Table 1). In the OVX rats, the BL expression of caspase 3 was comparable to that after six and 28d in the HA. On the other hand, a 1.4-fold significantly lower expression of caspase 3 was observed in the SCP at six days compared with the BL (Figure 3C, Table 1).

The levels of gene expression were compared between the HA- and SCP- augmented defects. At six days, defects filled with SCP revealed a 2.6-fold (*p = 0.4*) and 3.8-fold (*p < 0.05*) lower expression of TNF-α, in non-OVX and OVX respectively, compared with equivalents receiving HA (Figure 3A, Table 2). No significant differences in TNF-α expression were observed between SCP and HA at 28d, in either the non-OVX rats or the OVX rats. The expression of IL-6 was 3.2- (*p* = 0.1) and 2.2-fold (*p = 0.02*) higher, at six and 28d respectively, in non-OVX rat defects with SCP compared with those with HA. No differences were observed between SCP and HA for IL-6 expression in the OVX rats, at either six or at 28d (Figure 3B, Table 2). The caspase 3 expression level at six days was significantly lower, 1.8-fold, in non-OVX rat defects which received SCP compared with those that received HA (Figure 3C, Table 2). 

**Table 2:** Fold differences in gene expression.

	**OVX^*a*^/Non-OVX^*b*^**					**SCP^*a*^/HA^*b*^**			
	**BL**	**HA**		**SCP**		**Non-OVX**		**OVX**	
	**0d**	**6d**	**28d**	**6d**	**28d**	**6d**	**28d**	**6d**	**28d**
TNF-α	-1.3	1.6	1.6	1.1	1	-2.6	1.4	-3.8*	-1.2
IL-6	-1.4	1.8	1.5*	-1.4	-1.2	3.2	2.2*	1.1	1.3
Caspase3	-1.7*	-2	1.3	-1.4	1.1	-1.8*	-1.1	-1.3	-1.2
ALP	-2.7*	-2.9*	1.5	-2.3	1.2	-2	-1.1	-1.6	-1.4
Col1a1	1.15	-3.1*	1.3	-2.4	1.2	-1.8	-1.1	-1.4	-1.2
OC	-1.2	-1.6	1.8	-3	1.4	-1.5	-1.1	-2.7	-1.4
OPG	-1.9	-2.6	1.3	-1.4	-1.5	-1.8	1.3	-1.1	-1.5
RANKL	-1.5	-1.2	1.2	1.1	-1.1	-1.2	-1.1	1.1	-1.4
CR	-1.8	-1.5	1.3	1.1	1.9	-2.7*	-2.8*	-1.7	-1.9
CatK	-2.5*	-1.1	1.8*	1.1	-1.2	-1.6	-1.2	-1.3	-2.5*
VEGFA	-2.5*	2	1.3	-1.2	-1.1	1.6	1.4	-1.6	1

The Table shows (*left*) the difference between OVX and non-OVX rats at baseline (BL) and with each material at each time point and (*right*) the differences between SCP and HA in non-OVX and OVX rats at each time point.

Positive values indicate higher expression in (**^*a*^**) than in (**^*b*^**) whereas negative values indicate higher expression in (**^*b*^**) than in (**^*a*^**). Statistically significant differences are indicated by asterisks (*p < 0.05).

#### 4.2 Gene expression of bone formation, bone resorption and vascularisation markers

**Figure 4:**
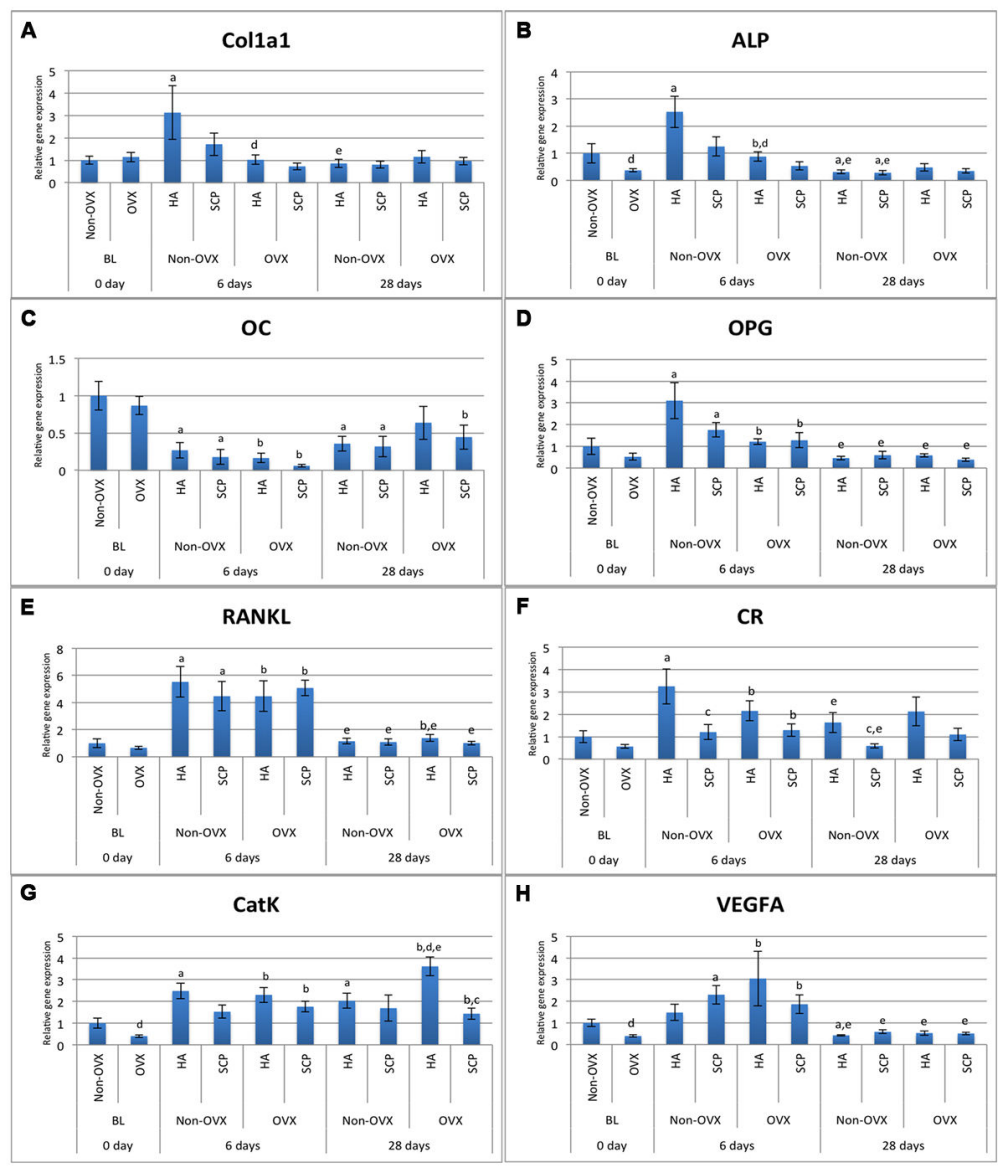
Gene expression analysis of bone formation, bone resorption and angiogenesis markers. The analysis was performed on the tissue harvested from defects filled with hydroxyapatite (HA) and strontium-doped calcium phosphate (SCP) in non-OVX and OVX rats after six and 28d of implantation. Expression levels of Col1a1 (A), ALP (B), OC (C), OPG (D), RANKL (E), CR (F), CatK (G) and VEGFA (H) Statistically significant differences (p < 0.05) are indicated by the small letters: a = significant difference between baseline non-OVX and non-OVX (HA or SCP; 6 or 28d); b = significant difference between baseline OVX and OVX (HA or SCP; 6 or 28d); c = significant difference between HA and SCP (non-OVX or OVX; 6 or 28d); d = significant difference between non-OVX and OVX (HA or SCP; 6 or 28d); e = significant difference between six days and 28d (HA or SCP; non-OVX or OVX). The results are presented as the mean ± SEM.

The baseline gene expression and the temporal changes in gene expression were evaluated. In both the non-OVX and OVX, the expression profile of the early bone formation marker ALP in HA defects was 2.5 times significantly higher at six days compared with BL (Figure 4B, Table 1). A similar observation was made for Col1a1 but only in the non-OVX rats (3.1-fold) (Figure 4A, Table 1). During this time period (6d), the expression of both markers in SCP defects (non-OVX and OVX) was comparable to BL expression levels (Figure 4A, B, Table 1). From six to 28d, the higher levels of Col1a1 in the HA defects decreased significantly, 3.6-fold (Figure 4A, Table 1), and, as a result, all the groups at 28d revealed levels comparable to those registered at BL (Figure 4A, Table 1). ALP expression revealed a higher level of reduction from six to 28d, where, at 28d, the non-OVX rats showed significantly lower levels, for both materials, compared with BL (Figure 4B, Table 1). The expression profile of the late bone formation marker, OC, was generally lower in all groups at six days when compared with BL. The expression of OC increased slightly for all groups from six to 28d, but the levels were still lower when compared with BL (Figure 4C, Table 1). The expression of the osteoclast key differentiation factor, RANKL (mainly expressed by osteoblasts), at six days, showed a significant five-fold upregulation for all groups, HA, SCP, non-OVX and OVX, compared with BL. From six to 28d, the RANKL expression was significantly downregulated for all groups and showed levels comparable to those at BL. However, at 28d, a higher expression of RANKL was still observed, but only in the HA OVX group compared with BL (Figure 4E, Table 1). The expression patterns of the osteoclast surface marker (CR) and osteoclast activity marker (CatK) were characterised by upregulated expression after six days for all groups apart from the SCP in the non-OVX (Figure 4F, G, Table 1). The same pattern was maintained for CatK after 28d, with an even higher level in the HA defects in the OVX rats compared with six days and BL (1.6- and nine-fold, respectively; *p < 0.05*) (Figure 4G, Table 1). The temporal expression of CR revealed a significant decrease from six days to 28d for both materials in the non-OVX rats, whereas no major changes were observed in the OVX rats (Figure 4F, Table 1).

The temporal gene expression of vascularisation marker VEGF revealed a general trend towards higher expression at six days compared with BL and a significant reduction from six days to 28d (Figure 4H, Table 1).

The levels of gene expression were compared between the HA- and SCP- augmented defects. In the non-OVX rats, the gene expression of osteoclast receptor CR was significantly reduced in the SCP compared with the HA, about 2.8-fold, at both six and 28d. A similar trend for CR expression was observed in the OVX rats, with lower expression in SCP compared with HA (Figure 4F, Table 2). However, the difference was not statistically significant. On the other hand, the osteoclast activity marker, CatK, showed a significant downregulation in SCP compared with HA, mainly in the OVX group at 28d. No major differences were found between HA and SCP groups with respect to the expression of Col1a1, ALP, OC, OPG, RANKL and VEGFA, regardless of time points or whether or not the rats were ovariectomised (Figure 4A-E, H).

### 5 Histology and histomorphometry

#### 5.1 Histology

**Figure 5:**
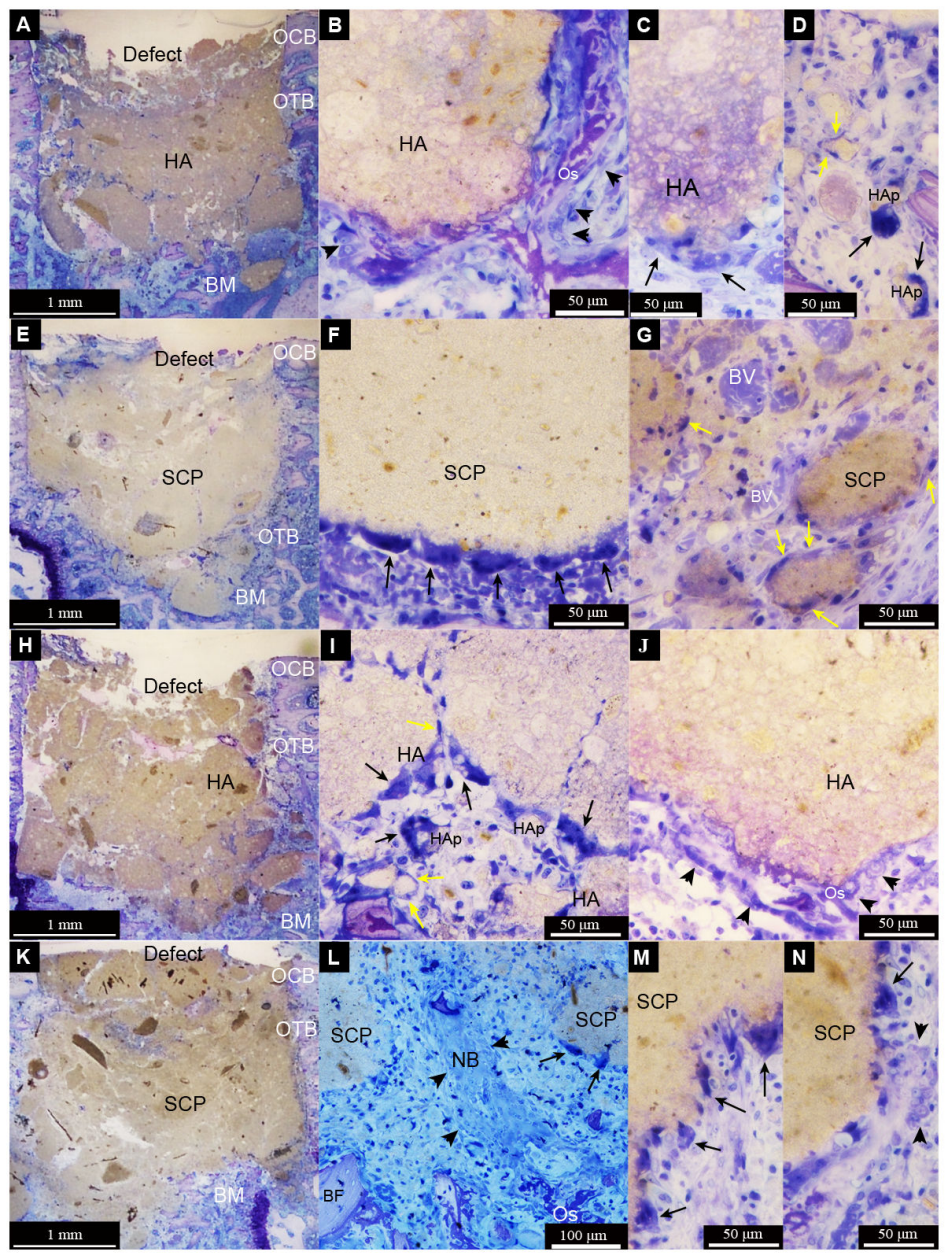
Light micrographs of non-decalcified toluidine blue-stained ground sections of femur defects after six days of healing. The defects were created in non-ovariectomised (non-OVX) (A-G) or ovariectomised (OVX) (H-N) rats. The defects were filled with either hydroxyapatite (HA) (A-D and H-J) or strontium calcium phosphate (SCP) (E-G and K-N) granules. The survey images for each group (A = non-OVX HA, E = non-OVX SCP, H = OVX HA, K = OVX SCP) show that the granules are generally within the defect boundaries in the trabecular area of the femoral epiphysis. During this time period, no major difference in the amount of granules was observed between the four different groups. However, a more defined shape of the HA granules is observed in contrast to the SCP, which have a homogeneous appearance with less well defined individual granules. All the defects appear to be largely occupied by the granules in contact with the original bone and bone marrow (BM) at the periphery of the defect. No evident bone formation at this magnification level is observed. The cut borders of the original cortical bone layer (OCB) and the original trabecular bone (OTB) are still clearly visible, with minimum signs of remodelling or outward bone formation. In the higher magnification images, multinucleated giant cells (indicated by black arrows) were very commonly detected at the surface of larger granules. In the HA groups, the multinucleated cells appear to be surrounding some smaller particles in the micron range (HAp) (D and I). Spindle-shaped mononuclear cells (macrophage-like cells; some are indicated by yellow arrows) are also detected in relation to granules and particles in all treatment groups (D, G and I). At high magnification, signs of bone formation are detected with osteoblast seams (indicated by black arrowheads) forming osteoid (Os) appearing directly on some HA granules (B and J) and at some distance from or in between the SCP granules (N). Numerous blood vessels (BV) are observed, especially in the SCP group (G).

After six days of implantation, both HA and SCP granules occupied the major portion of their defects (Figure 5A, E, H, K). The HA had a granular appearance and showed a relatively higher degree of particle dissociation, with both large- and small-sized particles (Figure 5A, H). The SCP had a relatively homogeneous appearance, with the bulky granules localised in the centre and the smaller granules gradually dissipating peripherally (Figure 5E, K). Granulation tissue was primarily observed filling the spaces between the granules in all groups. Inflammatory infiltrates were observed in the intergranular spaces, irrespective of the materials or OVX (Figure 5D, G, I). One important observation during this time period was the detection of multinucleated giant cells (MNGCs) that were frequently encountered in both the HA and SCP and irrespective of OVX or non-OVX. The MNGCs in the HA group appeared in association with both large- and small-sized granules (Figure 5C, D, I), whereas, in the SCP groups, MNGCs were mostly located at large bulky granules (Figure 5F, L, M, N). The process of vascularisation was evident, with new blood vessel formation, more commonly noted in SCP-filled defects (Figure 5G). Early signs of intramembranous osteogenesis were observed during this time period (6d), with osteoblast seams and osteoid observed in close proximity to both granule types and with no major differences between the OVX and non-OVX (Figure 5B, L). 

**Figure 6:**
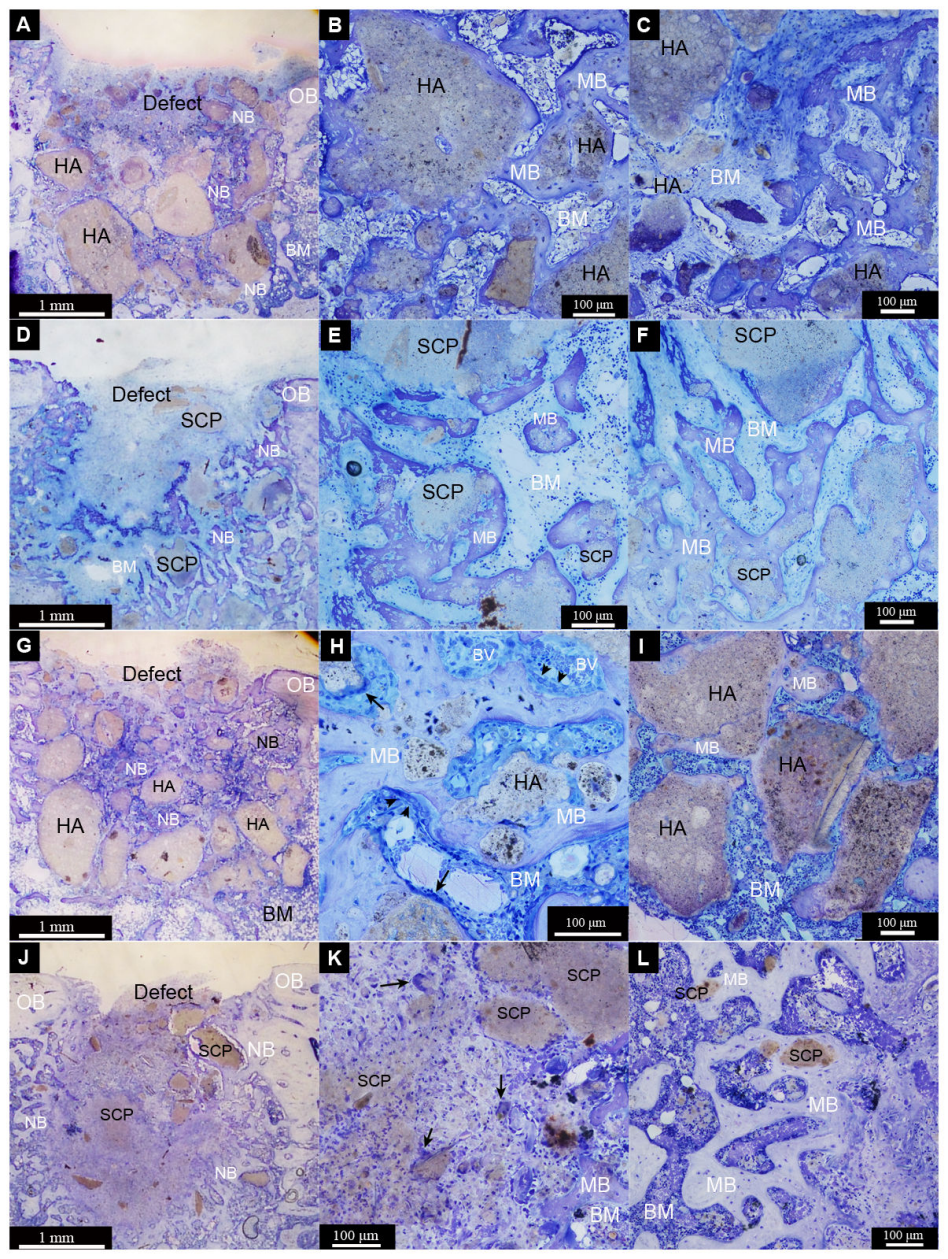
Light micrographs of non-decalcified toluidine blue-stained ground sections of femur defects after 28d of healing. The defects were created in non-ovariectomised (A-F) or ovariectomised (G-L) rats. The defects were filled with either hydroxyapatite (HA) (A-C and G-I) or strontium calcium phosphate (SCP) (D-F and J-L). The survey images for each group (A = non-OVX HA, D = non-OVX SCP, G = OVX HA, J = OVX SCP) show the pattern of new bone formation (NB) and the distribution of the remaining granules within the defect boundaries. An evident amount of separated granules with various sizes and shapes appears within the HA defects (A and G) and, at least at this magnification, new bone (NB) and bone marrow areas (BM) are more visible in the SCP defects (D and J). The demarcation line between the old bone (OB) and the newly formed bone (NB) at the defect borders is sometimes hardly defined (A and G), but it is well defined in some defects (D). (B, E, H and K) show that the granules are surrounded and interconnected by mature bone (MB) in the central region of the four different defects. A considerable amount of mature bone has formed in the central region of HA defects (B and H), in contrast to the central region of SCP defects (E and K). Multinucleated giant cells (some indicated by black arrows) were detected at the granule surface (H and K). (C, F, I and L) show that the granules are also surrounded and interconnected by mature bone in the peripheral region of the four different groups. The peripheral region of the HA defects are largely occupied by the remaining granules with less mature bone (C and I) and a more mature bone area in the peripheral region of SCP defects (F and L).

**Figure 7:**
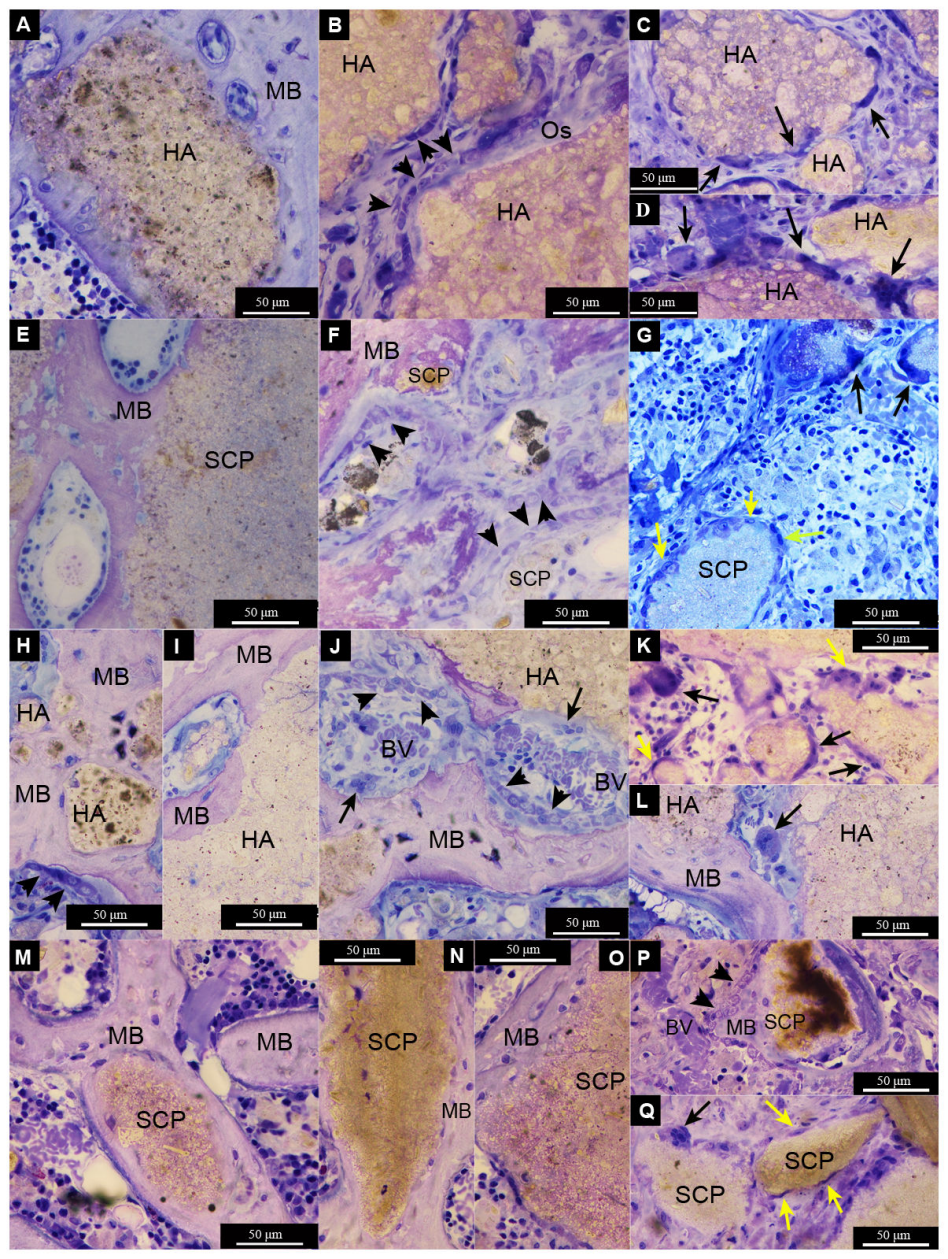
Light micrographs of non-decalcified toluidine blue-stained ground sections of femur defects after 28d of healing. The defects were created in non-ovariectomised (A-G) or ovariectomised (H-Q) rats. The defects were filled with either hydroxyapatite (HA) (A-D and H-L) or strontium calcium phosphate (SCP) (E-G and M-Q). Mature bone (MB) is formed around and in contact with the granule surface in the four different groups (A = non-OVX HA, E = non-OVX SCP, H and I = OVX HA, M = OVX SCP). Bone formation is clearly visible, with osteoblast seams (indicated by black arrowheads) forming osteoid (Os) appearing directly on some HA granules (B), while mature bone (MB) lined with osteoblasts is observed on the SCP granules (P). A series of osteoblast seams is clearly observed enclosing some granule remnants in the SCP non-OVX defect (F). In the HA OVX defect, signs of bone remodelling are obvious, with the formation of multinucleated cells, blood vessels (BV) and osteoblast seams between the mature bone and granule surface (J). Multinucleated giant cells (indicated by black arrows) were commonly detected at the surface of HA granules (C, D, K and L) and, to a lesser extent, on the SCP granules (G and Q). Spindle-shaped mononuclear cells (macrophage-like cells) (some are indicated by yellow arrows) were also detected in all groups (G, K and Q).

After 28d of implantation, both HA and SCP materials were still evident. The HA revealed granules of varying size but with a more distinct shape (Figure 6A, G). On the other hand, the SCP was dissipated in the defect area with few distinguishable granules mainly localised at the periphery of the defect (Figure 6D, J). During this period, a considerable amount of mature, well mineralised bone had formed. Bone was formed directly on the HA and SCP materials or within the intergranular spaces bridging the neighbouring granules (Figure 6B, E, H). Many granules of both types appeared circumferentially integrated in the formed bone. Two major differences between HA and SCP groups were determined. Firstly, a higher proportion of bone was detected at the centre of the HA defects (Figure 6B, H) whereas a larger proportion of bone was distributed at the periphery of the defects filled with SCP (Figure 6F, L). Secondly, the interface between bone and the HA granule surface had a clear demarcation line but with no evident separation observed at the magnification used (Figure 7A, H, I). In the SCP, the boundary was less easily distinguished, with a gradual diminution of the bone contrast towards the granule surface and conversely (Figure 7E, M). Another important observation after 28d was the prevalence of multinucleated cells in association with both material types. In the HA group, and irrespective of OVX or not, the MNGCs appeared on the surfaces of large granules, as well as apparently in the process of engulfing smaller granules (Figure 7C, D, K, L). Other multinucleated cells appeared on the surfaces of granules, bone and/or the interface zone between granule and bone and these had a more osteoclast-like phenotype with close proximity to osteoblast seams on surfaces of newly secreted osteoid (Figure 7J). The osteoclast-like cells were mostly restricted to the HA-filled defects and were seldom found in the SCP. In general, fewer MNGCs were observed during this period in the SCP-filled defects as compared to the HA.

#### 5.2 Histomorphometry

**Figure 8:**
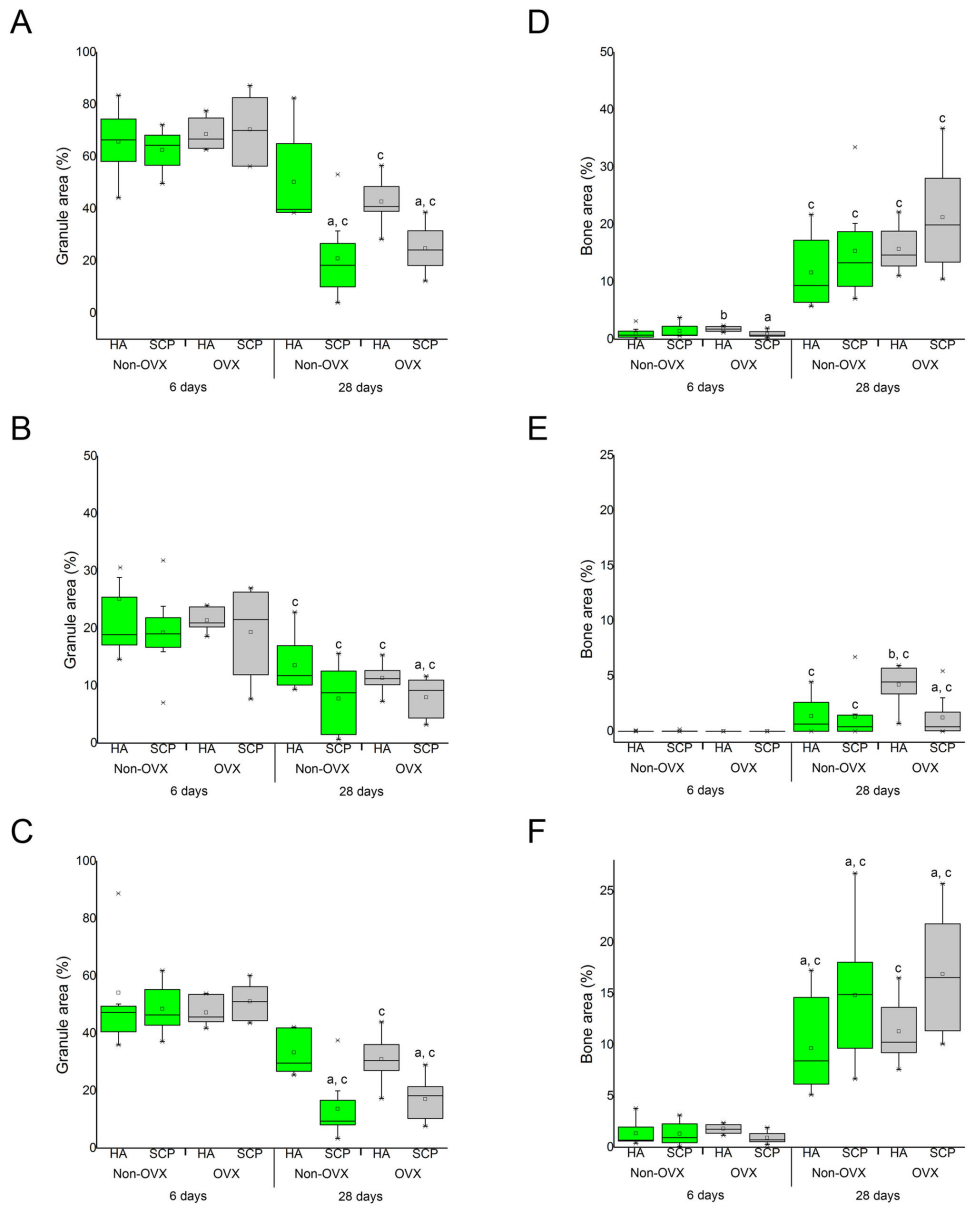
Histomorphometric analysis. The graphs show the granule (left) and bone (right) area percentages (%) after six and 28d of implantation. Percentages (%) of granule and bone areas were calculated at total (A, D), central (B, E) and peripheral (C, F) levels of the defects. Each box plot shows the mean, median, standard deviation and the range of measurements (n = 8). Statistically significant differences (p < 0.05) are indicated by the small letters: a = significant difference between HA and SCP (at each time point for each animal group); b = significant difference between non-OVX and OVX (at each time point for each material group); c = significant difference between six and 28 d (for each group).

In general, a low percentage of mineralised bone was recorded at six days (1-1.8% of the total defect area) and it had increased significantly at 28d for all groups (Figure 8D). After 28d, the total bone area percentages were 11.6 ± 2.3 (HA-non-OVX), 15.3 ± 3 (SCP-non-OVX), 15.7 ± 1.4 (HA-OVX) and 21.2 ± 3.2 (SCP-OVX) (Figure 8D). The measurements showed a trend towards higher total bone percentages in SCP compared with HA and in the OVX versus the non-OVX, although the differences were not statistically significant (Figure 8D). When the bone percentages were measured separately in the central (Figure 8E) and peripheral (Figure 8F) regions of the defects, statistically significant differences were found between the HA and SCP, as well as between the OVX and non-OVX rats, particularly at 28d. The topological measurements at 28d revealed a significantly higher percentage of bone area at the periphery of the defect treated with SCP granules both in the non-OVX (14.8 ± 2.2) and in the OVX (16.9 ±2.0) compared with the corresponding HA ones (9.6 ± 1.7 and 11.2 ± 1.1, respectively) (Figure 8F). At the same time, and only in the OVX rats, the central region of HA-treated defects had a significantly higher bone percentage (4.2 ± 0.6) compared with the SCP group (1.2 ± 0.6) (Figure 8E). The bone percentage in the central region of HA-treated defects in OVX rats was significantly higher than in the equivalent region in the non-OVX rats (1.3 ± 0.6) (Figure 8E).

The percentage of total granule area, shown in (Figure 8A), revealed comparable levels among all the groups after six days of implantation, which ranged between 63-70% of the total defect area. At 28d, the percentages of granule area had decreased significantly, with a higher level of reduction in SCP compared with HA and no major difference between OVX and non-OVX. In both non-OVX and OVX, the percentages of granule area dropped to a range of 21-25% (in the SCP) and 43-50% (in the HA) (Figure 8A).

## Discussion

Hydroxyapatite and strontium-substituted calcium phosphate granules were inserted in trabecular bone defects with the objective of evaluating bone healing and regeneration. The main emphasis was placed on the effect of strontium and the influence of the condition of the recipient bone, in this case as a result of ovariectomy. Although both materials showed comparable overall bone formation, the two materials exhibited major differences in the topological distribution of the formed bone and in degradation rates *in vitro* and *in vivo*. Further, the *in vivo* gene expression revealed that the two materials elicited different molecular activities, at least in those crucial for inflammation and osteoclastic functions. In addition, the total bone formation in the defects treated with the two materials did not differ significantly in association with OVX, at least within the time frame evaluated in this study.

A main finding was the differential distribution of the formed bone in association with the two materials. This observation was, at least in part, influenced by the OVX condition. When SCP granules were used, a larger amount of mineralised bone was observed in the defect periphery, irrespective of OVX or not. In contrast, the bone area in the central part of the defect was higher in conjunction with HA treatment, mainly in the OVX rats. The spatial difference in bone formation can be attributed to the difference in the degradation rates and the distribution of the degraded materials and ions. This postulation is supported by the *in vitro* degradation test and the *in vivo* histomorphometric analysis showing that the SCP granules were associated with higher degradation and dissolution than the HA. A somewhat simplified explanation is that the more degradable SCP granules provided less stable surfaces, which may not favour osteogenesis in the central region of the defect. It was shown, for example, that more degradable beta-TCP granules are associated with more peripheral bone formation, whereas more stable granules, consisting of a mixture of octacalcium and alpha calcium phosphate, showed more bone centrally [26].

Given that strontium has anti-osteoclastic potential, the present results corroborate previous results in which hydroxyapatite granules were mixed with an anti-resorptive drug, alendronate, and applied in similar defects in OVX rat femoral epiphyses [27]. In that study, Canettieri and co-authors found a closure of the bone defect after 21d using the HA alone, while, in the defects filled with alendronate-treated granules, no newly formed bone was found in the central area [27]. The authors suggested that the alendronate dose inside the defect may jeopardise the repair of the bone defect centrally, but, when molecules of the drug scattered and reached more distant sites, they may have favoured subperiosteal bone formation. In the present study, although no measurements were made of the spatial distribution of strontium at the different sites of the defect, it is possible that the *in vivo* dissolution of the SCP granules resulted in different concentrations of strontium peripherally and centrally. Supported by previous *in vivo* [28] and *in vitro* [29] findings showing a multiphasic and dose-dependent effect of systemically administered strontium on bone formation and mineralisation, it is possible that, in the present study, the concentration of strontium at the periphery of the defect was more optimal for bone formation than that localised in the centre. Furthermore, it is also possible that the local inhibition of the osteoclastic/remodelling activities results in a site-specific effect on bone regeneration. The present release data demonstrated a similar range of Sr (0.001 – 0.1 mM) as reported by Schumacher and co-authors [30] to promote the proliferation and differentiation of mesenchymal stem cells *in vitro*. Moreover, an inhibitory effect on osteoclast differentiation and activity was also observed at similar range of strontium concentrations [31]. In the present study, although the amount of released Sr, *in vitro*, was comparable to those promoting pro-osteoblastic [30] and anti-osteoclastic [31] effects, only an anti-osteoclastic effect was observed *in vivo*, as judged by gene expression analyses and histology. Whether the varying solubility of HA and SCP granules and/or the anti-resorptive effect of strontium had the essential influence on the different bone formation pattern of the two materials requires further investigation.

Strontium is generally believed to have a dual action of promoting osteogenic bone formation and inhibiting osteoclastic bone resorption. Strontium induced a higher expression of osteoblastic genes ALP, OC and bone sialoprotein, combined with increased bone nodules, and a reduction in the number of mature osteoclasts *in vitro* [19]. Similar observations were found when strontium was combined with biomaterials [32]. From a mechanistic point of view, the prevailing hypothesis about the strontium effect was supported, at least in part, by the present *in vivo* gene expression assays. This particularly applies when comparing the strontium effect on the expression of osteoclastic markers, where, in both animal groups, the defects filled with SCP displayed a lower expression of the osteoclastic surface receptor, CR, and, similarly, a lower trend for the osteoclastic activity marker, CatK. The gene expression data corroborate the histological observations, demonstrating less osteoclast-like cells in the SCP defects compared with HA. On the other hand, SCP did not evoke any major stimulatory effect on the expression of osteogenic differentiation genes. Further, on the morphological level, the overall bone formation did not differ between SCP and HA. The present findings relating to bone formation in association with SCP granules are therefore not in agreement with recent observations of higher expression of bone formation markers in the peri-implant bone of the Sr-incorporated titanium implants [33].

In order to explore the mechanisms by which strontium substitution would affect the bone healing process, we analysed the *in vivo* expression of major genes important for inflammation, bone formation and remodelling, angiogenesis and apoptosis. The observations relating to the inflammatory cytokines TNF-α and IL-6 indicate a diverse inflammatory effect by the two materials at least at gene expression level. The present data show that HA granules exclusively upregulate the TNF-α expression in both the OVX and non-OVX rat, but only during the early period of six days after implantation. On the other hand, SCP granules exclusively upregulate IL-6, during both early and late time periods, but mainly in the non-OVX. Since no major histological differences were observed on the level of inflammatory infiltrate between the two materials and/or the OVX and non-OVX, the TNF-α expression could be implicated in the recruitment of osteoclast precursors and their differentiation to mature osteoclasts in the HA defects. This is supported by the observation that HA treated sites were associated with higher expression of the osteoclast surface marker, CR, and the osteoclast differentiation marker, CatK. In fact, osteoclast differentiation requires an essential signal from the RANKL, expressed by the osteogenic cells, on which strontium has been suggested to act. For instance, previous *in vitro* data showed that strontium ranelate stimulates osteoblast maturation and also reduces the RANKL expression by the osteoblasts [34]. In this *in vivo* study, the RANKL expression appeared to be highly, yet equally, expressed in all defects with the presence of either SCP or HA. It is therefore possible that the increased expression of TNF-α in the HA defects promoted a higher osteoclastic differentiation compared with that in the SCP. Binding of TNF-α to TNFR1 on the osteoclast surface could augment the RANK-RANKL pathway [35] and may even promote osteoclast formation independently of RANKL, through other pathways [36,37].

Although IL-6 has been implicated in osteoclast differentiation and activity [38-40], the present study did not reveal a positive association between IL-6 expression and osteoclast appearance in any of the defects. Instead, a higher IL-6 expression in the SCP-treated defects paralleled a lower expression of the osteoclast surface marker, CR, in the same defects. IL-6 has multifunctional effects, not only in acute phase reactions and immune responses but also in bone remodelling [41-43]. For instance, Yoshitake and co-authors described a direct inhibitory effect of IL-6 on osteoclast progenitors, suppressing their differentiation by regulating the transcription of specific genes related to the MAPK system [44]. It can therefore be postulated that the *in vivo* osteoclastic responses in the calcium phosphate-based bone substitutes, with and without strontium, involve the differential regulation of specific cytokines with either a positive or a negative impact on the differentiation and activity of the osteoclast cells. Needless to say, in addition to the presence of strontium, this might be largely influenced by the material solubility and the *in vivo* degradation, the size of degraded particles, together with other parameters, which may determine the cellular response to the calcium phosphate ceramics [45].

Multi-nuclear giant cells (MNGCs) were also associated with the SCP granules; however, they lacked the osteoclastic histological characteristics in terms of proximity to bone remodelling sites (resorption lacunae, osteoblast seams and newly formed osteoid). The composition and the solubility of bioglass-based particles have been shown to determine whether MNGCs of the foreign body type or osteoclast type will predominate in bone defects [46]. Foreign body type MNGCs were detected on the surfaces of silicon-rich remnants, whereas osteoclast-like cells were detected on the bioglass particles after transformation in calcium phosphate shells [46]. However, the aforementioned study also linked the increased solubility of the particles to the higher predominance of the osteoclastic type of the MNGCs. Based merely on solubility, our findings are not in agreement, as the more soluble SCP revealed a relatively less osteoclastic type compared with the less soluble HA granules.

The *in vitro* analysis showed that both materials are degradable and that HA granules would resorb at a slower rate than the SCP. *In vivo*, the two materials were implanted in an attempt to have equal volumes of each type in each defect. This could not be achieved technically, since the SCP appeared to condense more easily and therefore more of their granules were needed per defect in comparison to the HA. Despite this, the comparable net weight of each type was inserted. The histomorphometric results showed that, for both materials, the percentage of circumferential areas of remaining granules calculated at 28d was less than those at six days. This indicates that both materials underwent *in vivo* biodegradation in parallel with the early events of bone formation and remodelling. Furthermore, it was evident that SCP granules underwent greater *in vivo* degradation during the present evaluation period. The present findings relating to the granules are comparable with recent results using scaffolds, where the degradation of strontium-substituted calcium phosphate scaffolds was shown to be higher than that of the HA ones after implantation in rabbit ulna segmental defects [47]. The biodegradation of calcium phosphate-based materials is generally believed to be dissolution driven, but it is also affected by cell-mediated processes [48-50]. Furthermore, the biomaterial dissolution in biological fluid is influenced by several properties such as crystal size, crystallinity and the porosity. In this study, the higher crystallinity of the HA is probably a major factor responsible for the observed lower degradation rate compared with SCP.

The early upregulation of the early osteogenic differentiation markers, ALP and Col1a1, was detected in the non-OVX rats compared with the OVX. The upregulation was significant in the HA defect and did not reach statistical significance in the SCP group, albeit with the same trend. Nevertheless, this transient upregulation during the six-day period did not match the histological bone formation picture, either in the non-OVX or in the OVX. Firstly, the HA group which displayed higher bone formation in the central region at 28d was the OVX one. Secondly, the SCP-treated defects were associated with greater peripheral bone formation both in the non-OVX and in the OVX. Although it was not possible to find a clear explanation for this discrepancy, the results are comparable to previous findings on osseointegration in non-OVX and OVX rats [10]. In the latter study, bone-related gene expression and histomorphometry of bone formed in T-shaped titanium chambers revealed comparable levels between non-OVX versus OVX rat after 28d of healing [10]. The present study shows that the OVX rats have a downregulation of osteogenic genes during the first six days of healing in defect augmented with bone substitute material. However, after 28d, they show a higher bone fraction in the central region of the defect when treated with less resorbable HA granules and a higher bone fraction in the peripheral region of the defect when treated with more resorbable, SCP granules. Taken together, ovariectomy impairs the early stage of osseointegration and bone regeneration, whereas at later stage a structural restitution is achieved. 

One interesting finding in the present study was the lower expression of OC in the augmented defects compared to the baseline expression. The exact explanation of this observation is not available. This may indicate an active steady-state bone remodelling, especially in trabecular bone sites. Given the lack of information about constitutive gene expression in normal and OVX rats, such possibility can not be excluded. Another possibility is that higher peaks for OC expression in the defects, augmented with HA or SCP, are attained at time points different from the evaluated time points in this study (for instance, after 3 and/or 14d). Furthermore, although we found an increased bone formation in association with either material at 28d, this was not in parallel with an increased OC expression. The observation on the increased bone formation at 28d was site-specific where HA showed higher bone formation in the centre whereas the SCP demonstrated a greater degree of bone formation in the periphery of the defect. It is possible that the areas with no or with minimum bone formation (periphery or centre, respectively) are also associated with low expression of OC. Since we have analysed the gene expression in the whole defect site, including periphery and centre, a site-specific variation in OC expression could have been masked. Site-specific sampling and gene expression analysis (e.g. using laser microdissection) may provide additional tool to resolve such assumption. 

## Conclusions

It is concluded that HA and SCP granules result in comparable overall bone formation in trabecular bone defects. As judged by gene expression and histological analyses, the two materials induced different inflammatory and bone remodelling responses. The modulatory effects are associated with differences in the spatial distribution of the newly formed bone.

## Supporting Information

Appendix S1 **Enzyme-linked immunosorbent assay (ELISA) and gene expression analyses comparing non-OVX and OVX animals.**
(DOCX)

Figure S1 **Enzyme-linked immunosorbent assay of rat serum samples.** The graphs show the levels of IL-1β (A), OC (B) and TRAP (C) in rat serum collected after six and 28d of implantation in ovariectomised (OVX) and non-ovariectomised (non-OVX) rats. Statistically significant differences (*p < 0.05*) are indicated by the small letters: **a** = significant difference between non-OVX and OVX; **b** = significant difference between six days and 28d. The results are presented as the mean ± SEM.
(TIF)

Table S1 **Ion contents in HA and SCP granules (µg/mg of granule).**
(DOCX)
