# Supplementary material for: Strontium-Doped Calcium Phosphate and Hydroxyapatite Granules Promote Different Inflammatory and Bone Remodelling Responses in Normal and Ovariectomised Rats
Source: PLoS One. 2013 Dec 23;8(12):e84932. doi: 10.1371/journal.pone.0084932 (PMC3871578; doi:10.1371/journal.pone.0084932)
Supplement: Appendix S1 — Enzyme-linked immunosorbent assay (ELISA) and gene expression analyses comparing non-OVX and OVX animals. (DOCX) [file pone.0084932.s001.docx]

**Appendix S1. Supporting information**

**Strontium-doped calcium phosphate and hydroxyapatite granules promote different inflammatory and bone remodelling responses in normal and ovariectomised rats**

Carina Cardemil^1,2,3,#,*^, Ibrahim Elgali^1,4,#^, Wei Xia^3,4^, Lena Emanuelsson^1,4^, Birgitta Norlindh^1,4^, Omar Omar^1,4^, Peter Thomsen^1,4^

^1^Department of Biomaterials, Institute of Clinical Sciences, Sahlgrenska Academy at University of Gothenburg, Gothenburg, Sweden

^2^Department of Oral and Maxillofacial Surgery, Örebro University Hospital, Örebro, Sweden

^3^Applied Materials Science, Department of Engineering Sciences, Uppsala University, Uppsala, Sweden

^4^BIOMATCELL VINN Excellence Center of Biomaterials and Cell Therapy, Gothenburg, Sweden

^#^ Equal contribution

*^*^Correspondence to:* Carina Cardemil; e-mail: carina.cardemil@biomaterials.gu.se

Phone: + 46 (0) 19 602 2193; Fax: + 46 (0) 19 602 3135

Department of Biomaterials, Institute of Clinical Sciences

Sahlgrenska Academy at University of Gothenburg

Box 412; SE-405 30; Gothenburg, Sweden

**Supporting Materials and Methods**

Enzyme-linked immunosorbent assay (ELISA)

The collected blood was centrifuged and frozen at -80ºC. An enzyme-linked immunosorbent assay (ELISA) was used to measure the serum levels of the proinflammatory cytokine interleukin-1beta (IL-1β), (from R & D Systems Europe Ltd, Abingdon, Oxon, UK) and of bone formation and remodelling proteins, osteocalcin (OC) and tartrate-resistant acid phosphatase (TRAP), (both from IDS Nordic a/s, Herlev, Denmark).

**Supporting Results**

Enzyme-linked immunosorbent assay (non-OVX vs. OVX)

After six days, but not after 28d, the OVX rats had a significantly higher serum level of IL-β than the non-OVX rats (Figure S1 A). Between six days and 28d, the serum level of OC had decreased significantly in both the OVX and non-OVX groups (Figure S1 B). The OVX rats showed a significantly lower level of TRAP compared with the non-OVX rats after both six days and 28d (Figure S1 C).

Comparative gene expression of proinflammatory and apoptosis markers between non-OVX and OVX

Whereas no significant differences were detected when comparing the BL expression of TNF-α and IL-6 between the non-OVX and OVX (Figure 3A, B, Table 2), the BL expression of caspase 3 was significantly lower, 1.7-fold, in the OVX compared with the non-OVX (Figure 3C, Table 2). In the defects filled with HA, the OVX rats revealed a 1.8-fold (*p = 0.1*) and 1.5-fold (*p = 0.01*) higher expression of IL-6 compared with the non-OVX rats after six and 28d, respectively (Figure 3B, Table 2). On the other hand, both inflammatory cytokines showed no significant difference between non-OVX and OVX, in either time period, when the defects received SCP.

Comparative gene expression of bone formation, bone resorption and vascularisation markers between non-OVX and OVX

At BL, the OVX rats revealed a significantly lower expression of ALP, CatK and VEGFA, about 2.5-fold, compared with the non-OVX rats (Figure 4B, G, H, Table 2). At six days, the HA- and SCP-filled defects revealed lower expression levels of Col1a1 and ALP, three-fold (*p < 0.05*) and 2.5-fold (*p = 0.07*) respectively, in the OVX rats compared with the non-OVX rats (Figure. 4A, B, Table 2). The bone formation markers did not reveal major differences after 28d between OVX and non-OVX, in either the HA or the SCP. At 28d, the osteoclastic marker, CatK, showed a significant 1.8-fold upregulation in the OVX compared with the non-OVX, mainly in the HA-treated defects (Figure. 4G, Table 2).
