# Supplementary figures and images for: Strontium-Doped Calcium Phosphate and Hydroxyapatite Granules Promote Different Inflammatory and Bone Remodelling Responses in Normal and Ovariectomised Rats
Source: PLoS One. 2013 Dec 23;8(12):e84932. doi: 10.1371/journal.pone.0084932 (PMC3871578; doi:10.1371/journal.pone.0084932)

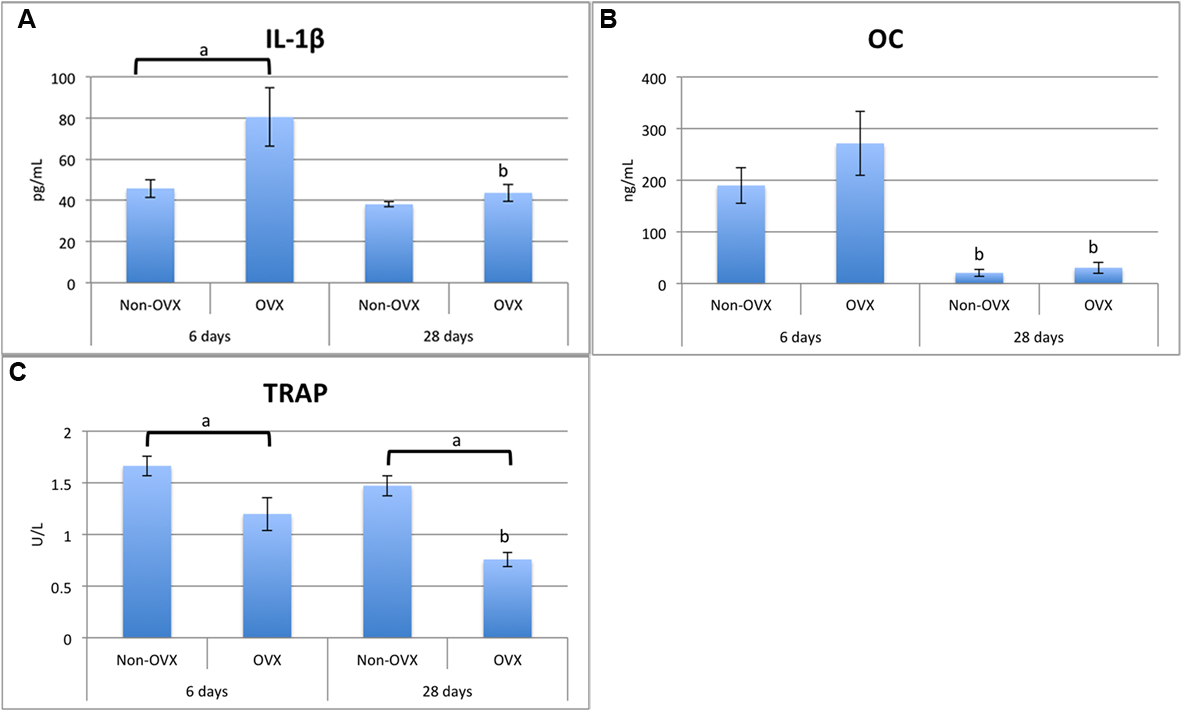

Supplement: Figure S1 — Enzyme-linked immunosorbent assay of rat serum samples. The graphs show the levels of IL-1β (A), OC (B) and TRAP (C) in rat serum collected after six and 28d of implantation in ovariectomised (OVX) and non-ovariectomised (non-OVX) rats. Statistically significant differences (p < 0.05) are indicated by the small letters: a = significant difference between non-OVX and OVX; b = significant difference between six days and 28d. The results are presented as the mean ± SEM. (TIF) [file pone.0084932.s002.tif]
