# Supplementary material for: Strontium-Doped Calcium Phosphate and Hydroxyapatite Granules Promote Different Inflammatory and Bone Remodelling Responses in Normal and Ovariectomised Rats
Source: PLoS One. 2013 Dec 23;8(12):e84932. doi: 10.1371/journal.pone.0084932 (PMC3871578; doi:10.1371/journal.pone.0084932)
Supplement: Table S1 — Ion contents in HA and SCP granules (µg/mg of granule). (DOCX) [file pone.0084932.s003.docx]

Table S1. Ion contents in HA and SCP granules (µg/mg of granule)

|  | **Ca** | **Mg** | **P** | **Sr** | **Sr/Ca** |
| --- | --- | --- | --- | --- | --- |
| HA | 358.5 | 1.7 | 188.8 |  |  |
| SCP | 132.4 | 23.5 | 173.1 | 276.5 | 0.96 |
